# *Staphylococcus aureus *and autoimmune uveitis reactivation in childhood: a possible correlation?

**DOI:** 10.1186/1824-7288-35-34

**Published:** 2009-11-12

**Authors:** Paolo Mora, Erilda Kamberi, Francesca Manzotti, Laura Zavota, Jelka G Orsoni

**Affiliations:** 1 Institute of Ophthalmology, Azienda Ospedaliero - Universitaria, Parma, Italy; 2 Division of Pediatrics, Azienda Ospedaliero - Universitaria, Parma, Italy

## Abstract

The role of infectious agents in autoimmune diseases has been the subject of several studies and is still under investigation. Here a paediatric case series of autoimmune uveitis is reported. An exacerbation of the ocular inflammation occurred in concomitance with nasal colonisation by *Staphylococcus aureus*.

## Introduction

Sir,

Infectious agents are believed to play a role in the pathogenesis of some autoimmune diseases. Among the most investigated micro-organisms are Streptococci and Herpes viruses [1-3]. An abnormal T cell response to low levels of some *Staphylococcus aureus *superantigens was demonstrated in vitro in Behçet's disease (BD) [4]. Similar correlations have also been reported in vivo. The relationship between nasal *Staphylococcus aureus *carriage and Wegener granulomatosis (WG) has been demonstrated [5]. Moreover, exacerbation of the clinical signs of BD due to Staphylococcal gingival infection has been described. Not only were oral ulcers aggravated, but also genital ulcers and skin lesions worsened. Elimination of the bacterial infection resulted in complete recovery [6]. In the following report, we describe three paediatric cases that tested positive for nasal *Staphylococcus aureus *concomitantly with the reactivation of their autoimmune ocular inflammation.

## Case Report

Three boys, aged between 8 and 11 years, were referred to our Service for severe ocular inflammation with progressive visual impairment. Clinical, haematological and instrumental evaluations allowed to exclude an infectious origin of the inflammation, which was eventually diagnosed as bilateral idiopathic pars planitis in two cases, and unilateral posterior uveitis with papillitis in one case. Systemic immune-modulating treatment resulted in satisfactory control of the ocular disease, with visual recovery in 15-30 days. Nasal swab specimens obtained pre-treatment, while ocular inflammation was still active, tested negative for bacterial colonisation in all cases.

The three young patients experienced a relapse of their ocular inflammation after approximately 45, 150 and 180 days, respectively. Nasal swab culture was repeated at the time of reactivation and tested positive for *Staphylococcus aureus *in all cases, in the absence of any respiratory symptoms. Immune-modulating treatment was temporally increased and associated with systemic antimicrobial therapy (lasting 7-10 days). Complete quiescence of the ocular inflammation was achieved after three weeks. At this time nasal swabs tested negative in 2/3 boys.

## Discussion

Several immunological mechanisms have been proposed to explain the relationship between some infectious agents and an active status of autoimmune diseases. In WG, genetic polymorphism of the receptor for the Fc fragment of immunoglobulins G (FcγR) may decrease *Staphylococcus aureus *clearance, thus promoting chronic carriage and resulting in preferential binding of FcγR to a subset of IgG-ANCA isotypes characterised by marked immunogenicity [7]. Furthermore, as significant sequence homology exists between the mammalian and microbial Heat Shock Proteins (50% homology), it has been suggested that bacterial HSP-responsive T cells stimulate autoreactive T cells by cross-reactivity mechanisms [1]. In fact, both antistreptococcal and antiretinal HSP60 antibodies were raised in the serum samples of patients with BD and uveitis [8]. Increased anti-HSP65 antibody responses were also present in the cerebrospinal fluid of patients with neuro-BD with parenchimal involvement [9].

We describe a small paediatric case series in which an extraocular *Staphylococcus aureus *infection may have acted as trigger for reactivation of autoimmune ocular inflammation. In these cases, nasal swabs proved useful to identify and treat the nasal bacterial carrier status of the young patients. A recent series stated that the mean rate of nasal *Staphylococcus aureus *colonisation in childhood is 36%, showing an age-related parabolic distribution with peak incidence at the age 11 years [10]. This corresponds to the age range in our patients. A larger number of cases, however, is necessary to evaluate if the elimination of nasal staphylococcal colonisation, even if asymptomatic, may help control the autoimmune ocular inflammation.
